# Homicide and geographic access to gun dealers in the United States

**DOI:** 10.1186/1471-2458-9-199

**Published:** 2009-06-23

**Authors:** Douglas J Wiebe, Robert T Krafty, Christopher S Koper, Michael L Nance, Michael R Elliott, Charles C Branas

**Affiliations:** 1 Department of Biostatistics and Epidemiology, University of Pennsylvania School of Medicine, Philadelphia PA, USA; 2 Department of Statistics, University of Pittsburgh, Pittsburgh PA, USA; 3 Police Executive Research Forum, Washington DC, USA; 4 Department of Surgery, The Children's Hospital of Philadelphia, Philadelphia PA, USA; 5 Department of Biostatistics, University of Michigan, Ann Arbor MI, USA

## Abstract

**Background:**

Firearms are the most commonly used weapon to commit homicide in the U.S. Virtually all firearms enter the public marketplace through a federal firearms licensee (FFL): a store or individual licensed by the federal government to sell firearms. Whether FFLs contribute to gun-related homicide in areas where they are located, in which case FFLs may be a homicide risk factor that can be modified, is not known.

**Methods:**

Annual county-level data (1993–1999) on gun homicide rates and rates of FFLs per capita were analyzed using negative binomial regression controlling for socio-demographic characteristics. Models were run to evaluate whether the relation between rates of FFLs and rates of gun homicide varied over the study period and across counties according to their level of urbanism (defined by four groupings, as below). Also, rates of FFLs were compared against FS/S – which is the proportion of suicides committed by firearm and is thought to be a good proxy for firearm availability in a region – to help evaluate how well the FFL variable is serving as a way to proxy firearm availability in each of the county types of interest.

**Results:**

In major cities, gun homicide rates were higher where FFLs were more prevalent (rate ratio [RR] = 1.70, 95% CI 1.03–2.81). This association increased (p < 0.01) from 1993 (RR = 1.69) to 1999 (RR = 12.72), due likely to federal reforms that eliminated low-volume dealers, making FFL prevalence a more accurate exposure measure over time. No association was found in small towns. In other cities and in suburbs, gun homicide rates were significantly lower where FFLs were more prevalent, with associations that did not change over the years of the study period. FFL prevalence was correlated strongly (positively) with FS/S in major cities only, suggesting that the findings for how FFL prevalence relates to gun homicide may be valid for the findings pertaining to major cities but not to counties of other types.

**Conclusion:**

Modification of FFLs through federal, state, and local regulation may be a feasible intervention to reduce gun homicide in major cities.

## Background

Homicide is a major cause of death in the U.S. and the second leading cause of death among 15–34 year-olds.[1] During each year of the past quarter century (1980–2005), more homicides were committed with firearms than with all other weapon types combined.[2] Firearms accounted for 331,270 homicides over this period.[2]

A correspondingly large firearm manufacturing and dealer distribution system exists in the U.S. and is thought to contribute to the incidence of firearm homicide. [3-5] The number of federally-licensed firearm dealers (FFLs), which include gun stores and individuals that are licensed by the federal government to ship, transport, and receive firearms in interstate commerce and engage in retail sales, may play a major role. There were 104,840 FFLs in the U.S. in 2001.[6] No publicly available data report the number of guns sold by FFLs. However, data on gun purchases and on guns seized and guns recovered by police indicate that firearms flow through FFLs into U.S. communities at a rate that sums into the millions each year. [7-9] A state-level study, using self-reports of household firearm ownership as a proxy for gun availability, found that the states where guns were most common experienced the highest rates of gun homicide.[10] Similarly, it is possible that a greater number of FFLs is associated with greater gun availability and ultimately, increased gun homicide rates.

Although the federal government is responsible for regulating which individuals or businesses are issued an FFL, the decision about where an FFL can operate is a local matter. Therefore, it is helpful to adopt an urban planning perspective when considering the possibility that FFLs could be impacting local homicide rates.[11,12] Like other businesses, FFLs are subject to regulations including zoning laws which dictate how land parcels can be used. In addition to zoning laws, state-level legislation, although currently in place in only a minority of states, can mandate additional licensing requirements and periodic inspection of gun dealer records.[13] Therefore, if FFLs do act as a spigot through which firearms flow into a community and thereby contribute to homicide, it is possible that regulating the locations and activities of stores where firearms are sold is a way to curb homicide. With this in mind, we considered whether licensed gun dealers function as a proxy for gun availability in counties in the U.S., and studied whether having a disproportionately high number of FFLs in a county was associated an elevated rate of homicide committed with guns. Other studies examining mortality as a function of gun availability have used FS/S – the proportion of suicides that were committed with firearms as opposed to other methods – as a way to measure by proxy the extent of gun availability in a geographic area. Whereas FS/S appears to serve well as a proxy for gun availability, FS/S is not a risk factor that is modifiable.[10,14,15] FFLs are modifiable, in contrast, in terms of their locations and retail practices, and thus in this way this analysis is investigating a public health issue with direct policy relevance.

## Methods

### Data and Variables

Our dataset consisted of seven separate, year-specific entries for 3,112 counties (including the District of Columbia and county equivalents such as boroughs and independent cities), totaling 21,784 observations of county-level data for the U.S. from 1993 to 1999. The outcome measure was annual firearm homicide rates per 100,000 population in U.S. counties for the years 1993 through 1999. The rates were calculated with firearm homicide data from the National Center for Health Statistics' (NCHS) Multiple Cause of Death files (defined as International Classification of Diseases-Ninth Revision [ICD-9] codes E965.0–E965.4 for 1993–1998 and ICD-10 codes X93–X95 for 1999) and population data from U.S. Census data. Permission to include counties with fewer than 100,000 persons was obtained from the NCHS Division of Vital Statistics. We calculated this outcome measure separately by year for each U.S. county. Our primary predictor measure was the annual per capita prevalence of "type one" (firearm dealer) and "type two" (pawnbroker) FFLs (per 1,000 population) in counties for the years 1993 through 1999. The FFL data were obtained from Basic Information Systems, Inc. (Wheaton, Maryland), which provided data from the Bureau of Alcohol, Tobacco, Firearms and Explosives (ATF) on the annual number and type of FFLs in the U.S. by county for the study period. FFL data were not available for counties in Alaska and therefore Alaska was not included in the analysis. FFL data for years after 1999 were not available.

Covariates were used to control for several county-level factors thought to be potential confounders of the association between FFL rates and homicide rates being studied (e.g., [10]): percent of population 15–29 years old, percent male, percent African American, percent Native American, percent Hispanic, percent married, percent living alone, percent female headed households, average per capita income, percent of persons below the poverty level, percent of the civilian labor force unemployed, percent over age 25 who were college educated, hospital beds per capita, and percent of arrests that were drug-related. Each of these variables was obtained from the U.S. Census Bureau and the Area Resource File[16] with the exception of drug-related arrests, which was obtained from the Federal Bureau of Investigation's county-level Uniform Crime Reports (UCR),[17] and was incorporated given evidence that drug arrests may be feasible as an indicator of drug activity.[18,19] Because the UCR arrest data are derived from police jurisdictions, which do not correspond exactly with county boundaries, a second variable that adjusted for the discrepancy by distance-based weighting between police jurisdiction centriods and county centroids was included in the model. Also, the covariates included a variable for urbanization defined according to a modification of the rural-urban continuum classification (mRUC) scheme [20-22] designed by the U.S. Department of Agriculture.[23] This variable initially included 11 categories that were collapsed to four categories for parsimony during preliminary analyses: major cities (mRUC 1: central counties of one million population or more); other cities (mRUC 2: central counties of metropolitan areas of one million population or more); suburbs (mRUC 3–5: fringe counties of metropolitan areas of one million population or more, ranging to counties in metropolitan areas of fewer than 250,000 population); and small towns (mRUC 6–11: urban counties with a population of 20,000 or more adjacent to a metropolitan area, ranging to completely rural counties of less than 2,500 urban population not adjacent to a metro area). Each variable was measured annually except for urbanization, persons below the poverty level, female-headed households, persons living alone, persons married, and persons college-educated. These variables were based on data available either decennially and/or for certain intercensal years. Intercensal years without data were linearly interpolated or forecast based on known values. Additionally, a linear trend for year was included to account for temporal variability.

Given that the availability of firearms in one county may depend on how many FFLs exist in surrounding counties, a variable was included to reflect the prevalence of FFLs in counties surrounding the index county, weighted by the inverse of the squared rectilinear distance between county population-weighted centriods. The leniency of state firearm laws in each county was controlled for as well. This was done by coding each county according to a classification scheme published annually since 1997. [24-29] The scheme uses an integer scale ranging from 0 to 100, with 0 representing maximum restrictiveness and 100 representing maximum leniency, to reflect state laws pertaining to the acquisition, ownership, and transportation of firearms and ammunition. Leniency scores were assigned separately to each county for each year. The leniency scores used for 1997 through 1999 were taken directly from published information. We linearly extrapolated leniency scores for the years 1993 through 1996 based on the values published for the period 1997 through 2002. Finally, a variable containing county-level annual nongun homicide rates (defined as ICD-9 codes E960–E964 and E965.5–E967.9 for 1993–1998 and ICD-10 codes X85–X92 and X96–Y08 for 1999) was derived for inclusion in the final statistical model. This was done to explore the possibility that such an adjustment could help control for a latent homicidal tendency at the county level and isolate the contribution of FFLs to gun homicide.

### Statistical Analysis

Plots were generated to assess annual prevalence rates of FFLs and rates of homicide in the U.S. Negative binomial generalized linear regression conducted at the county level was used to estimate the association between the prevalence of FFLs and rates of gun homicide in U.S. counties during the 1993–1999 study period. Model coefficients were converted into incidence rate ratios (RR), and the result of the final statistical model are presented. Correlation stemming from use of multiple data years was accounted for with generalized estimating equations under a working independence correlation matrix. Spearman correlation coefficients and variance inflation factors were used to identify multicollinearity and for diagnostic purposes. Also, variation by year and urbanization, for reasons discussed below, was then investigated by restricting analyses to individual data years and using interaction terms to permit the relation between the prevalence of FFLs and rates of gun homicide to vary across counties according to their size (i.e., on the rural-urban continuum). The variable used to represent a linear trend for year was excluded from the year-specific models. The adjusted RR estimates derived from these models are presented in a summary table.

To gain insight into how well the FFL prevalence variable may be serving as a way to proxy gun availability in counties of each grouping, the proportion of suicides committed with firearms (FS/S) – which is considered an accurate proxy for household gun availability and has been studied in state-level and region-level analyses [10,14,15] – was computed within each county by year and modeled in place of our FFL variable. Spearman correlation coefficients were also calculated to determine how well FS/S was correlated with the FFL prevalence rate within counties of each grouping.

## Results

**Figure 1:**
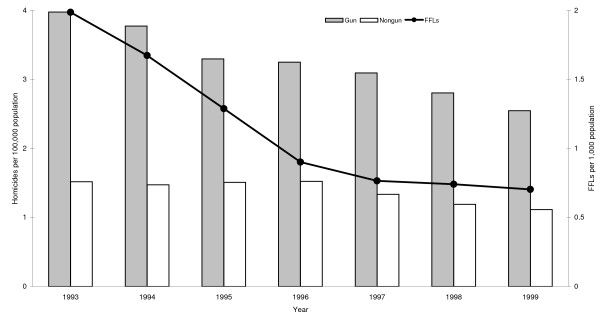
**Homicide incidence rate and federal firearms licensee (FFL) prevalence rate in the U.S., 1993–1999**.

In 1993, 17,984 gun homicides and 6,141 nongun homicides occurred in the U.S. At that time a total of 253,314 gun manufacturers, gun stores and individuals held active licenses to sell firearms (i.e., FFLs). The annual incidence rates of gun and nongun homicide and the annual prevalence rates of FFLs in the U.S. from 1993 to 1999 are shown in Figure 1. Rates of gun homicide dropped considerably over this period, ranging from 3.98 per 100,000 in 1993 to 2.55 per 100,000 in 1999. By contrast, rates of nongun homicide fluctuated little, ranging from a high in 1993 of 1.52 per 100,000 to a low in 1999 of 1.11 per 100,000. The prevalence of FFLs decreased even more dramatically than did gun homicide, ranging from 1.99 per 1,000 in 1993 to 0.70 per 1,000 in 1999. The average amount by which gun homicide rates and FFL prevalence rates decreased over the seven-year period was approximately equal (23% and 22%, respectively).

Table 1 shows characteristics of U.S. counties. The rate of gun homicide ranged from 0.00 per 100,000 to 187.27 per 100,000 and the rate of nongun homicide ranged from 0.00 per 100,000 to 97.75 per 100,000, with average gun and nongun homicide rates of 3.25 per 100,000 and 1.38 per 100,000, respectively. The number of FFLs ranged from 0.001 per 1,000 to 30.93 per 1,000 with an average of 1.15 per 1,000. There were no counties without FFLs; each county had at least one FFL active during each study year.

**Table 1:** Characteristics of U.S. counties, 1993–1999

	Mean	Standard deviation	Minimum	Maximum
Homicide rate (per 100,000)	4.63	7.87	0.00	187.27
Gun	3.25	6.53	0.00	187.27
Non-gun	1.38	3.54	0.00	97.75
Federal firearms licensees (FFL) (per 1,000)	1.15	1.10	0.001	30.93
% of population age 15–29 years	0.31	0.36	0.09	0.81
% male	49.32	1.95	43.86	82.20
% African American	9.20	15.00	0.00	86.76
% Native American	1.18	7.42	0.00	97.69
% Hispanic	5.21	11.81	0.00	99.41
% married	44.96	5.04	18.50	58.60
% living alone	24.67	3.83	7.91	77.51
% of female headed households	10.17	3.98	0.78	36.07
% of persons below poverty level	15.22	6.58	0.00	55.27
% of adult population college educated	24.23	7.06	8.41	64.36
% of arrests that were drug related	7.29	4.96	0.00	100.00
Average per capita income (%)	19347	4985	1185	75702
Unemployment rate	5.87	3.04	0.00	37.90
Hospital beds per 1,000 population	2.85	3.98	0.00	7.41
Urbanization (11-code mRUC)	8.00	2.74	1	11
Urbanization (collapsed mRUC)	3.67	0.61	1	4
Leniency of state gun laws	75.71	17.21	3	100

mRUC denotes modified rural-urban continuum codes. 11 codes were collapsed into 4 groups for analysis.

Data exclude Alaska due to lack of FFL information.

**Figure 2:**
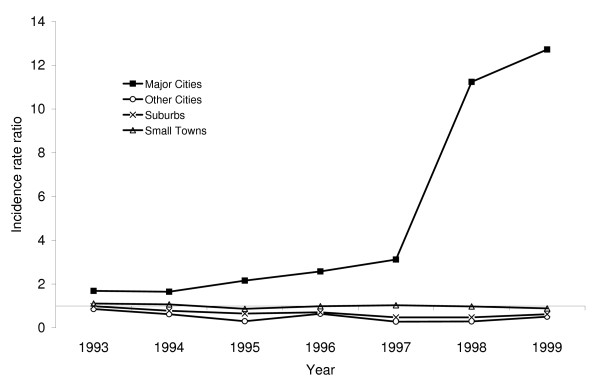
**Annual gun homicide rates as a function of the prevalence of federal firearms licensees (FFLs) in U.S. counties, by county type, 1993–1999**.

Table 2 shows results of the overall statistical model, which adjusted for covariates using regression but did not permit for the possibility that the relation between the prevalence of FFLs and gun homicide rates could vary over the years of the study period and according to county type (i.e., urbanization). The variable for percent of female headed households was excluded during the model building process due to multicolinearity. The results based on this overall model suggested that the prevalence of FFLs in U.S. counties was not associated with the rate of gun homicide in the county (RR = 0.98, 95% confidence interval [CI] 0.93 to 1.04) (Table 2). However, inclusion of an interaction term revealed that the relation between FFLs and gun homicide was found to vary significantly by urbanization (p < 0.01). The subsequent modeling revealed that in major cities, a disproportionately high prevalence of FFLs was associated with significantly higher gun homicide rates (RR = 1.70, 95% CI 1.03 to 2.81) (Table 3). Additionally, the magnitude of this association increased significantly over the study period with an average increase of 90% per year (p < 0.001) (Figure 2). By contrast, in other cities and in suburbs a disproportionately high prevalence of FFLs was associated with significantly lower gun homicide rates (RR = 0.73, 95% CI 0.57 to 0.93; RR = 0.87, 95% CI 0.77 to 0.97, respectively), and the magnitude of these associations did not change when tested for trend over the study period. The prevalence of FFLs was not associated with gun homicide rates in small towns. Inclusion of the covariate representing county-level rates of nongun homicide did not substantively change the results. Also, the results did not change substantively when the variable that was used to adjust for the impact of the prevalence of FFLs in surrounding counties was excluded from the models.

**Table 2:** Gun homicide rates as a function of the number of federal firearms licensees in U.S. counties

	Incident rate ratio	SE	P-value	95% CI
FFLs	0.98	0.027	0.482	0.93, 1.04
FFLs in surrounding counties	1.03	0.009	0.001	1.01, 1.05
Year	0.96	0.009	0.000	0.95, 0.98
% poverty	1.05	0.004	0.000	1.05, 1.06
% married	1.04	0.005	0.000	1.03, 1.05
% college educated	1.00	0.003	0.699	0.99, 1.01
% African American	1.03	0.001	0.000	1.03, 1.03
% Native American	1.01	0.003	0.006	1.00, 1.01
% Hispanic	1.01	0.002	0.000	1.00, 1.01
% 15–24 years old	0.95	0.432	0.913	0.39, 2.32
% male	1.02	0.009	0.075	1.00, 1.03
% living alone	1.02	0.006	0.000	1.01, 1.04
% of arrests that were drug related	1.02	0.003	0.000	1.01, 1.02
Drug arrest jurisdiction adjustment	0.92	0.037	0.030	0.85, 0.99
Hospital beds	1.00	0.000	0.000	1.00, 1.00
Average per capita income	1.00	0.000	0.096	1.00, 1.00
Unemployment rate	1.01	0.006	0.331	0.99, 1.02
Leniency of state gun laws	1.01	0.001	0.000	1.01, 1.01
Major cities	1.97	0.226	0.000	1.57, 2.46
Other cities	1.39	0.117	0.000	1.17, 1.63
Suburbs	1.27	0.045	0.000	1.19, 1.37
Small towns (reference)	--			

Results of generalized linear negative binomial regression.

SE denotes standard error.

CI denotes confidence interval.

**Table 3:** Gun homicide rates (per 100,000 population) as a function of the prevalence of federal firearms licensees (FFLs) (per 1,000 population) in U.S. counties, by county type and year, 1993–1999

	**Incident rate ratio** **(95% CI)**	**Incident rate ratio** **(95% CI)**			
		
	All counties	Major cities	Other cities	Suburbs	Small towns
1993–1999	0.98 (0.93, 1.04)	1.70 (1.03, 2.81)	0.73 (0.57, 0.93)	0.87 (0.77, 0.97)	1.00 (0.95, 1.05)
1993	1.09 (1.02, 1.16)	1.69 (0.98, 2.90)	0.86 (0.67, 1.11)	0.99 (0.84, 1.18)	1.11 (1.04, 1.18)
1993	1.03 (0.91, 1.15)	1.65 (0.72, 3.77)	0.62 (0.42, 0.92)	0.78 (0.63, 0.96)	1.07 (0.96, 1.19)
1995	0.84 (0.73, 0.94)	2.16 (0.65, 7.20)	0.30 (0.17, 0.53)	0.65 (0.52, 0.82)	0.87 (0.77, 0.98)
1996	0.95 (0.80, 1.13)	2.58 (0.33, 19.99)	0.64 (0.08, 5.10)	0.71 (0.47, 1.08)	0.99 (0.83, 1.17)
1997	0.96 (0.80, 1.15)	3.12 (0.22, 44.78)	0.28 (0.08,0.96)	0.48 (0.30, 0.76)	1.03 (0.87, 1.13)
1998	0.92 (0.73, 1.15)	11.24 (0.46, 277.38)	0.29 (0.10, 0.87)	0.48 (0.31, 0.72)	0.98 (0.79, 1.22)
1999	0.85 (0.67, 1.07)	12.72 (0.64, 253.19)	0.51 (0.13, 2.00)	0.62 (0.38, 1.01)	0.89 (0.70, 1.12)

Results of generalized linear negative binomial regression models adjusted for covariates.

CI indicates confidence interval.

The results of the models run with FS/S used in place of the FFL prevalence variable (not presented) were generally consistent with our findings, although the link between gun availability and gun homicide appeared to be even stronger (positive) and more widespread than seen in the analysis presented here. Importantly though, FS/S was strongly (positively) correlated with FFL prevalence in major cities (Spearman correlation coefficient = 0.67) but was weakly correlated and not correlated with FS/S in other cities (0.32), suburbs (0.16), or small towns (0.07), respectively. From this evidence it appears that FFL prevalence is a good proxy for firearm availability in major cities only, suggesting that the findings for how FFL prevalence relates to gun homicide may be valid for the findings pertaining to major cities but not to counties of other types.

## Discussion

Our analyses provide evidence of an association between the per capita rate of licensed firearm dealers in a county and its rate of firearm homicide. In particular, we found that having a disproportionately high number FFLs was associated with significantly higher rates of firearm homicide in major cities. As such, FFLs may represent a risk factor for gun homicide that is modifiable. To the best of our knowledge, the association between licensed gun dealers and homicide rates has not previously been estimated. We also found more FFLs to be associated with significantly lower firearm homicide rates in other cities and in suburbs. Possible explanations for both findings are discussed below.

Our evaluation strategy followed the assumption that the number of FFLs in a county gives some indication of the prevalence of firearms (i.e., a proxy of gun availability). Although no available data report the number of guns sold per dealer, the basic finding that major cities having the most FFLs per capita also have the highest rates of gun homicide is consistent with what is known about how FFLs and communities relate in terms of gun availability. Data from gun traces (a determination of the chain of ownership, usually conducted in connection with a criminal investigation) conducted by the ATF may provide the best insight. Between July 1996 and December 1998, the ATF conducted 1,530 trace investigations to determine whether guns used during crimes were trafficked from an FFL into the illegal gun market and to determine the point of first purchase.[8] FFLs accounted for less than 10 percent of the 1,530 trace investigations but for nearly half (40,000) of all firearms involved in these traces. The average number of firearms trafficked by the FFLs under investigation was 350, which far exceeds the average number of firearms trafficked by other means including gun shows (130 guns), unlicensed gun dealers (75 guns), and straw purchasers (37 guns). The large volume of firearms that can be obtained by FFLs is possibly what underlies this discrepancy and why FFLs may figure prominently as a risk factor. In addition, a study where researchers telephoned FFLs and posed as customers provides additional evidence of how FFLs can facilitate the flow of guns to criminals. Gun dealers were generally willing to sell a handgun even when the buyer indicated an intention to purchase the gun illegally on behalf of someone else.[30]

Other ATF data provide additional support for the possibility that gun homicide is a function of local FFLs. Guns are often found to have been used for criminal purposes not far from the gun dealer where they were first obtained. Approximately 62 percent of crime guns traced by the ATF were first purchased from FFLs in the state where they were recovered by police, one-quarter (25.9%) of crime guns were recovered in the county where they were purchased, and 10.5 percent were recovered in a county adjacent to the county of purchase, almost all of which were in the same state (9.5%).[6] Moreover, almost one-third (32.2%) of traced crime guns are recovered by police within 10 miles of the FFL where they were first purchased, and over one-third (34.3%) are recovered between 11 and 250 miles of the FFL where they were first purchased.[6,8] Thus, an FFL appears most likely to have an effect in the home or surrounding counties.

A recent study showing that gun dealers in or near major cities are at substantially elevated risk of selling guns used in crime may help to explain the strong positive association found here between FFL prevalence and gun homicide in major cities specifically.[31] Also, we found that the association between FFLs and gun homicides in major cities grew stronger from 1993 to 1999. This finding is consistent with what resulted when in the 1990s the federal government took steps to regulate FFLs more closely. Before this time, the process to obtain a license to sell firearms was appreciably simpler.[4] The Gun Control Act of 1968 required the ATF to issue a license to any applicant who was at least 21 years old, had premises from which they intended to conduct business, and who otherwise was not prohibited by law from purchasing a firearm.[4] At the time, the fee to obtain or annually renew an FFL was $10. The ATF received an average of 33,000 applications for FFLs each year over the decade that followed. Fully 169,052 FFLs were active by 1978. That number increased steadily thereafter and by 1992 reached a national peak of 284,117 FFLs. Not all FFLs were legitimate businesses, however. Any FFL enabled the holder to purchase large numbers of firearms, often at wholesale prices, and to buy from sellers in other states.[6] Many of these dealers made few if any registered sales, suggesting they were not truly engaged in the business of firearms dealing as required by federal law, and a substantial proportion of the extant FFLs were not in compliance with applicable federal, state, and local laws.[4]

With the system becoming increasingly difficult for the ATF to monitor, Congress acted and in 1993 and 1994 increased the FFL application fee 20-fold to $200 and imposed new laws intended to shut down inactive or corrupt FFLs.[4] In the years that followed, the number of FFLs nationally dropped from about 260,000 in 1993 to 80,000 in 1999. Our effect estimates suggest that the association between FFL prevalence and homicide may have been weaker in the early 1990s than in later years because there existed a large number of low volume dealers who contributed less to the supply of firearms.[4] Hence, with FFLs over time becoming a better measure of the exposure under study, the actual magnitude of the association between FFLs and gun homicide may be more accurately portrayed in the last few years of our study.

In contrast to what was observed for major cities, we found a negative association between gun homicide and FFL prevalence in other cities and suburbs. When considered in conjunction with the finding that FFL prevalence and FS/S are correlated strongly in major cities but correlated weakly in other cities and suburbs, this suggests that FFL prevalence is not a good proxy for gun availability in other cities and suburbs and hence the models based on those areas should not be interpreted as providing valid estimates of the relation between gun availability and gun homicide. We can consider these findings in light of our understanding of how the relation between FFLs and gun homicide may vary across counties of different urbanization types. In major city areas with higher crime rates, there will be greater criminal demand for guns and, hence, a larger illegal market for guns. It thus seems more likely that a weapon sold in a major city, as compared to one sold in another county type, will end up in the hands of a criminal user through theft, straw purchase, gun trafficking, or some other kind of transaction in the secondhand market. Also, it is possible that handguns rather than long guns account for a higher share of guns sold in major cities. Further, it would also stand to reason that an "average" gun possessor has a greater chance of using a gun criminally in an area with higher rates of gun violence. Also, gun culture and the roles of guns in peoples' lives vary dramatically across urban-rural continuum. Firearm ownership is more widespread in rural areas than urban areas, so the need to purchase a firearm from an FFL may be less necessary in rural than urban areas. The role of firearms certainly varies by county type in terms of firearm mortality, in that rates of firearm-related mortality in the U.S. are equally high in both the most urban and the most rural counties, with the nuance being that it is the gun homicide rate that is high in the most urban counties and it is the gun suicide rate that is high in the most rural counties.[20] Each of these points highlights the importance of stratifying analyses by county type and identifying variables that measure firearm availability accurately in the county type at the focus of a particular study, an important point that has been made previously.[32] The FS/S comparisons suggest that the FFL variable used here provides an adequate proxy in major city counties alone.

If the FFL variable is not a good proxy for gun availability in counties we have defined as other cities and suburbs, our analyses cannot inform the issue of how gun availability relates to gun homicide in counties of these types. It may be the case that the impact of guns on a community may vary by community type and may be protective in other cities and suburbs. In one study of 170 U.S. cities with a population of at least 100,000, however, rates of homicide and of gun-related assault were found to be positively associated with the prevalence of firearms.[33] Even so, it is possible that the mixing in that study of what we have termed major cities, other cities and suburbs may have prevented the authors from detecting modification of this effect across area type. Another study of counties in Illinois initially found that the rate of firearm ownership was negatively associated with all measures of violent crime, including homicide, but had failed to control for urbanization.[34] Subsequent multivariate analyses with control for urbanization found no significant association between the rate of firearm ownership and homicide. A number of ecologic studies conducted at the state level [10,14] and individual-level studies [35-37] alike have found firearm availability to be a risk factor for homicide rather than a protective factor, yet other studies have not found clear effects of the relation between gun availability and homicide, e.g.,[34,38] and a recent National Academy of Sciences report concluded that the body of research on this topic is inconclusive.[39] Our findings highlight the need to account for urbanization in the studies that will follow.

Our analysis had several strengths. First, it was conducted at the county level to account for within-state variability in homicide rates and FFL prevalence. This also allowed us to control for the possibilities that the homicide rate in a county was influenced by FFLs in surrounding counties and by the leniency or permissiveness of neighboring state firearm laws. Second, our analysis examined the link between FFLs and homicide over county urbanization type. Third, the study years coincided with a period when changes to federal firearm licensing regulations produced a change in the composition of the pool of FFLs nationally. As FFLs became fewer the pool became more homogenous, and hence may have provided an exposure variable that became a better measure of gun availability over time. The finding that the association between FFLs and gun homicide in major cities grew stronger over time adds support to our interpretation of the results, as does the finding based on our comparison with FS/S that FFL prevalence appears to be a good proxy for gun availability in counties defined as major cities but not in other county types.

Also, two aspects of the present study are unique and have implications for how firearm homicide is studied and how the incidence of firearm homicide may be reduced. First, because the link between gun homicide and gun availability (as measured by the prevalence of FFLs) was found to vary significantly within states according to the urbanization levels of counties, it appears that studies conducted using broader geographic units of analysis (states, census divisions, etc.) may fail to detect important nuances in the nature of gun availability.

Second, to the best of our knowledge, our study, in focusing on gun dealers as a potential risk factor for homicide, is the first to assess a tangible measure of gun availability that can be modified as part of prevention activities. Law enforcement, city planners, and legal strategists in cities with high gun homicide rates can concretely focus in on excessive or problem gun dealers as opposed to the more nebulous issue of "gun availability." Moreover, local efforts to close down illegal gun commerce have already shown the potential to be effective.[33,40,41] As one example, zoning laws, which control the location and operation of stores and individual dealers licensed to sell firearms, have been used to regulate locations of FFLs in several U.S. communities.[11,12,42] An attempt to launch a coordinated effort to identify and act on problematic gun dealers will surely face challenges, however. For one, a key component of such efforts will be the policing activities of the ATF which, in having inspected fewer than 10% of FFLs in each year since 1979 and fewer than 5% of FFLs in most of those years, may have resources insufficient for the task.[6]

Our analysis also had limitations. As discussed, we could not account for the actual volume of firearms introduced by each FFL into the community. Although gun sales data are not currently available for a more focused test, the recent National Academy of Sciences report called for better information on FFLs to be collected and made available for research.[39] Additionally, we did not explicitly accommodate spatial autocorrelation in the estimation of the FFL effect estimates. Refitting the models presented in Table 2 and Table 3 including a simultaneous autoregressive (SAR) structure,[43] assuming that correlation declined in proportion to the square of the distance between counties, generally changed coefficients by less than 5%. Also, data for FFLs for years after 1999 were not available. Thus we could not analyze a more recent period. Nevertheless, we do not anticipate that the relation observed here between FFLs and homicide would have changed since the study period and thus this characteristic of the data should not be interpreted as devaluing the findings. Finally, it may be that high rates of homicide may lead to increased demand for firearms and hence additional FFLs, in which case the results of the "FFL as risk factor" hypothesis that our models have been designed to test would be spurious. A stronger analytic approach would be to test whether within-county increases in FFL prevalence were followed by increases in the rate of gun homicide. We considered that approach, but found many instances in which a county experienced no gun homicides in certain years but some homicides in the subsequent year, which prevents an annual change in homicide rate from being calculated. Also, as discussed above, we found evidence that FFL prevalence became a better proxy for firearm availability over time, which led to our preference for the 1999 models and our judgment to refrain from including change models in the current manuscript. An instrumental variable approach could be pursued as well, to attempt to remove from the models the influence of circularity that may exist. We hope this manuscript will inform how such design alternatives may be approached, and acknowledge their need given the cross-sectional nature of the present study.

## Conclusion

If locations of retailers licensed to sell firearms are indeed functioning as a spigot through which deadly firearms flow into criminal hands, then communities with greater geographic access to these dealers should ostensibly experience more firearm homicides. Our findings are consistent with the hypothesis that this is occurring in major U.S. cities. The modification of FFLs, as tangible entities that are tracked and overseen at the national level and, in some cases, at the state and local levels, may be a feasible intervention to reduce firearm homicide.

## Competing interests

The authors declare that they have no competing interests.

## Authors' contributions

CB, MN and ME conceived of the study and supervised all aspects of its implementation. ME and RK computed variables involved in the spatial component of the analysis and DW and ME conducted the statistical analysis. DW and CB drafted the manuscript. All authors helped to conceptualize ideas, interpret findings, and revise drafts of the manuscript.

## Pre-publication history

The pre-publication history for this paper can be accessed here:



## References

[B1] CDC (2008). WISQARS Leading cause of death reports, 2005. http://www.cdc.gov/injury/wisqars/.

[B2] Fox JA, Zawitz MW (2008). Weapons used (1980–2005). In: Homicide trends in the U.S. http://www.ojp.usdoj.gov/bjs/homicide/tables/weaponstab.htm.

[B3] Diaz T (1999). Making a killing: the business of guns in America.

[B4] Koper CS (2002). Federal legislation and gun markets: how much have recent reforms of the federal firearms licensing system reduced criminal gun suppliers?. Criminology and Public Policy.

[B5] Wintemute GJ (2000). Relationship between illegal use of handguns and handgun sales volume. JAMA.

[B6] ATF (2002). Firearms commerce in the United States.

[B7] Cook PJ, Ludwig J (1997). Guns in America: national survey on private ownership and use of firearms (NCJ 165476).

[B8] ATF (2000). Following the gun: enforcing federal laws against firearms traffickers.

[B9] PF (1996). Guns in America: results of a comprehensive national survey on firearms ownership and use.

[B10] Miller M, Azrael D, Hemenway D (2002). Rates of household firearm ownership and homicide across US regions and states, 1988–1997. Am J Public Health.

[B11] Dannenberg AL, Jackson RJ, Frumkin H, Schieber RA, Pratt M, Kochtitzky C, Tilson HH (2003). The impact of community design and land-use choices on public health: a scientific research agenda. Am J Public Health.

[B12] Corburn J (2004). Confronting the challenges in reconnecting urban planning and public health. Am J Pub Health.

[B13] Vernick JS, Hepburn LM, Ludwig J, Cook PJ (2003). State and federal gun laws: trends for 1970–1999. Evaluating gun policy.

[B14] Miller M, Azrael D, Hemenway D (2002). Firearm availability and suicide, homicide, and unintentional firearm deaths among women. J Urban Health.

[B15] Miller M, Azrael D, Hemenway D (2002). Firearm availability and unintentional firearm deaths, suicide, and homicide among 5–14 year olds. J Trauma.

[B16] Area Resource File (ARF), 1994–2000.

[B17] U.S. Dept. of Justice FBoI Uniform Crime Reporting Program Data [United States]: County-Level Detailed Arrest and Offense Data (1993 – 1999) (ICPSR study numbers 6545, 6669, 6850, 2389, 2764, 2910 and 3167).

[B18] Warner BD, Wilson Coomer B (2003). Neighborhood drug arrest rates: are they a meaningful indicator of drug activity? A research note. J Res Crime Delinquency.

[B19] Rosenfeld R, Decker SH Are arrest statistics a valid measure of illicit drug use? The relationship between criminal justice and public health indicators of cocaine, herion, and marijuana use. Justice Quarterly.

[B20] Branas CC, Nance ML, Elliott MR, Richmond TS, Schwab CW (2004). Urban-rural shifts in intentional firearm death: different causes, same results. Am J Public Health.

[B21] Nance ML, Denysenko L, Durbin DR, Branas CC, Stafford PW, Schwab CW (2002). The rural-urban continuum: variability in statewide serious firearm injuries in children and adolescents. Arch Pediatr Adolesc Med.

[B22] Branas CC, Richmond TS, Schwab CW (2004). Firearm homicide and firearm suicide: opposite but equal. Public Health Rep.

[B23] Butler MA, Beale CL (1994). Rural-urban continuum codes for metro and nonmetro counties, 1993.

[B24] Kappas JS (1997). Traveler's guide to the firearm laws of the fifty states.

[B25] Kappas JS (1997). Traveler's guide to the firearm laws of the fifty states (first edition, second printing).

[B26] Kappas JS (1998). Traveler's guide to the firearm laws of the fifty states (third edition).

[B27] Kappas JS (1999). Traveler's guide to the firearm laws of the fifty states (fourth edition).

[B28] Kappas JS (2000). Traveler's guide to the firearm laws of the fifty states (fifth edition).

[B29] Kappas JS (2002). Traveler's guide to the firearm laws of the fifty states (sixth edition).

[B30] Sorenson SB, Vittes KA (2003). Buying a handgun for someone else: firearm dealer willingness to sell. Inj Prev.

[B31] Koper CS (2007). Crime gun risk factors: buyer, seller, firearm, and transaction characteristics associated with gun trafficking and criminal gun use. Report to the National Institute of Justice.

[B32] Hemenway D (2004). Appendix A: Methodology. Private guns, public health.

[B33] Kleck G, Patterson E (1993). The impact of gun control and gun ownership levels on violence rates. Journal of Quantitative Criminology.

[B34] Bordua DJ, Byrne JM, Sampson RJ (1986). Firearms ownership and violent crime: a comparison of Illinois counties. The social ecology of crime.

[B35] Kellermann AL, Rivara FP, Rushforth NB, Banton JG, Reay DT, Francisco JT, Locci AB, Prodzinski J, Hackman BB, Somes G (1993). Gun ownership as a risk factor for homicide in the home. N Engl J Med.

[B36] Wiebe DJ (2003). Homicide and suicide risks associated with firearms in the home: a national case-control study. Ann Emerg Med.

[B37] Grassel KM, Wintemute GJ, Wright MA, Romero MP (2003). Association between handgun purchase and mortality from firearm injury. Inj Prev.

[B38] Centerwall BS (1991). Homicide and the prevalence of handguns: Canada and the United States, 1976 to 1980 [see comments]. Am J Epidemiol.

[B39] Wellford CF, Pepper JV, Petrie CV, Eds (2005). Firearms and violence: a critical review National Academy of Sciences, National Research Council.

[B40] Braga AA, Pierce GL (2005). Disrupting illegal firearms markets in Boston: the effects of Operation Cease Fire on the supply of new handguns to criminals. Criminology and Public Policy.

[B41] Webster DW, Bulzacchelli MT, Zeoli AM, Vernick JS (2006). Effects of undercover police stings of gun dealers on the supply of new guns to criminals. Inj Prev.

[B42] Veen J, Dunbar S, Reuland M, Steadman J (1997). The BJA Firearms Trafficking Program: demonstrating effective strategies to control violent crime (NCJ 166818).

[B43] Cressie NAC (1993). Statistics for spatial data.

